# 本草物质科学研究技术专辑引言

**DOI:** 10.3724/SP.J.1123.2026.06014

**Published:** 2026-08-08

**Authors:** Xinmiao LANG, Yanfang LIU, Xingya XUAN

## 引言

本草物质科学研究的核心目标是系统解析中药复杂的物质组成、作用靶点及其协同作用机制，从而建立一门对中药物质层面进行科学化、系统化、体系化研究的新兴学科。其研究逻辑在于：通过融合多学科交叉技术，深刻揭示中药复杂体系中“化学物质”与“生物功能”之间的内在关联，进而阐释多成分、多靶点协同调控的整体性作用规律。本研究领域致力于构建一个“源于临床，归于临床”的现代中药创新体系。该体系以标准化中药为基础，发展“靶点-通路-网络-疾病”关系明确的现代中药，并借助高质量的临床研究，推动其有效性与科学性获得广泛认可。

要从分子层面阐明中药科学原理，先进的分离和分析技术是关键前提。近年来，科研人员在中药分离分析、活性分子发现等技术领域取得一系列重要进展，有力推动了中药科学原理向分子层面的深入。为了集中展示我国学者在本草物质科学研究技术领域的最新成果，展望未来研究方向，受《色谱》期刊委托，我们组织了“本草物质科学研究技术专辑”，邀请了国内高等院校和科研院所等相关领域专家学者为本刊撰稿。

本专辑由1篇视角、4篇综述、6篇研究论文和1篇技术与应用组成，涉及中药分离纯化技术、中药色谱-质谱联用分析技术和中药多组学技术等研究领域。我们衷心希望通过本专辑的出版，充分展示我国学者在本草物质科学研究技术领域的最新成果，推动学科交叉与合作研究，为生命健康领域的发展贡献力量。

希望本专辑能吸引更多《色谱》读者的关注，为本草物质科学研究注入新的启发与思考，携手推动中药复杂物质体系分离分析技术的创新突破与学科交叉融合，助力中药传承创新与高质量发展。

衷心感谢各位作者及审稿专家的鼎力支持和奉献！

本专辑客座主编

中国科学院大连化学物理研究所

梁鑫淼 研究员

刘艳芳 研究员

薛兴亚 研究员

2026年6月7日

